# A case of successful cannulation for biliary enteric anastomosis stenosis via peroral cholangiopancreatoscope

**DOI:** 10.1055/a-2791-4497

**Published:** 2026-02-17

**Authors:** Yu Zou, Yinquan Pu, Ting Jiang, Yuefeng Hu, Xinhua Zhao, Xiaoan Li

**Affiliations:** 1 536557 Department of Gastroenterology, Mianyang Central Hospital, School of Medicine, University of Electronic Science and Technology of China, Mianyang, China; 2 536557 Hepatobiliary and Pancreatic Surgery, Mianyang Central Hospital, School of Medicine, University of Electronic Science and Technology of China, Mianyang, China; 3 536557 Endoscopy Center, Mianyang Central Hospital, School of Medicine, University of Electronic Science and Technology of China, Mianyang, China

**Fig. 1:**
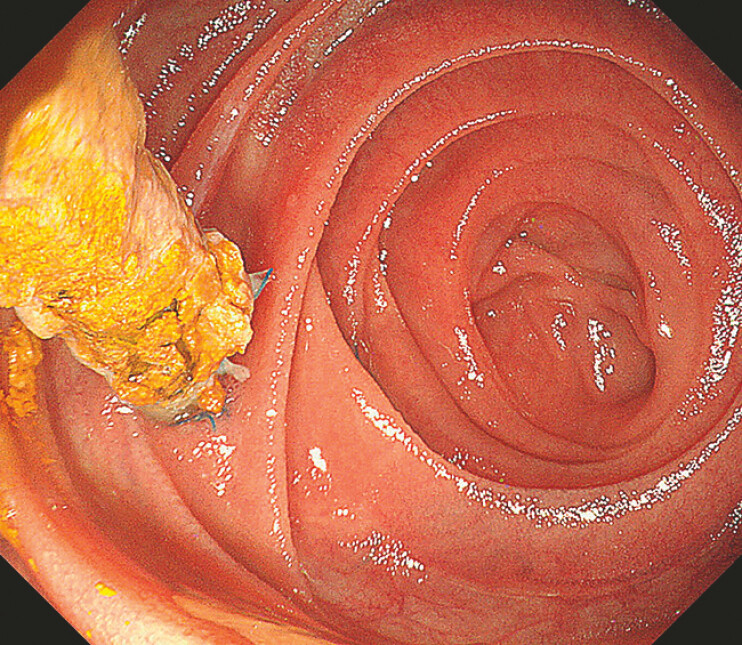
The endoscope was advanced to the region of the biliary-enteric anastomosis; the pancreatico-enteric anastomosis and the pancreatic duct stent were visualized, while the biliary-enteric anastomosis was not identified.

**Fig. 2:**
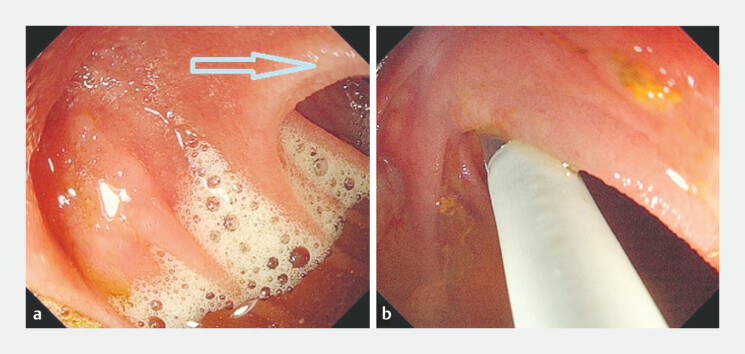
**a** It was suspected that the biliary-enteric anastomosis might be situated behind the mucosal folds. **b** The tip of the peroral cholangiopancreatoscope was advanced behind the mucosal folds.

**Fig. 3:**
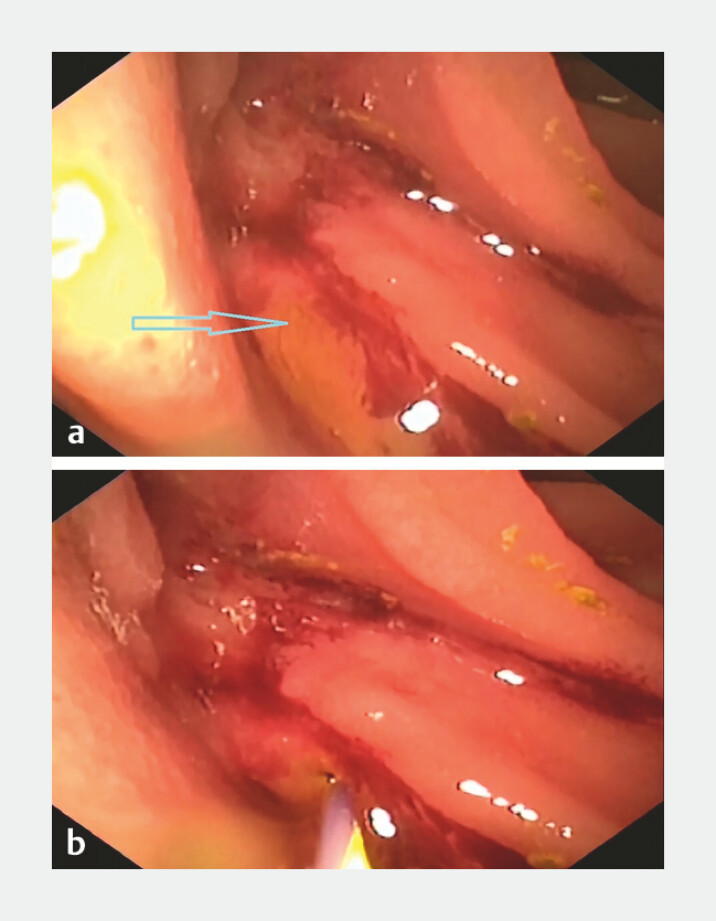
**a** A small orifice behind the mucosal folds was visualized on images obtained by the peroral cholangiopancreatoscope. **b** The guidewire was smoothly advanced through the small orifice into the bile duct.

**Fig. 4:**
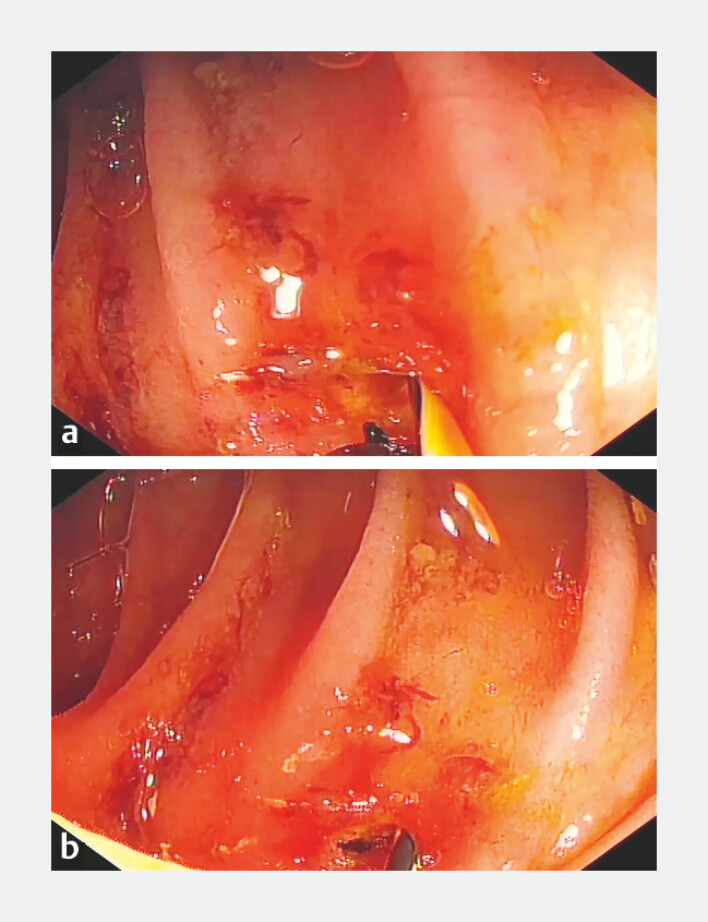
A needle knife was used to perform a precut of the biliary-enteric anastomosis: **a** before the precut procedure and **b** after the precut procedure.

**Fig. 5:**
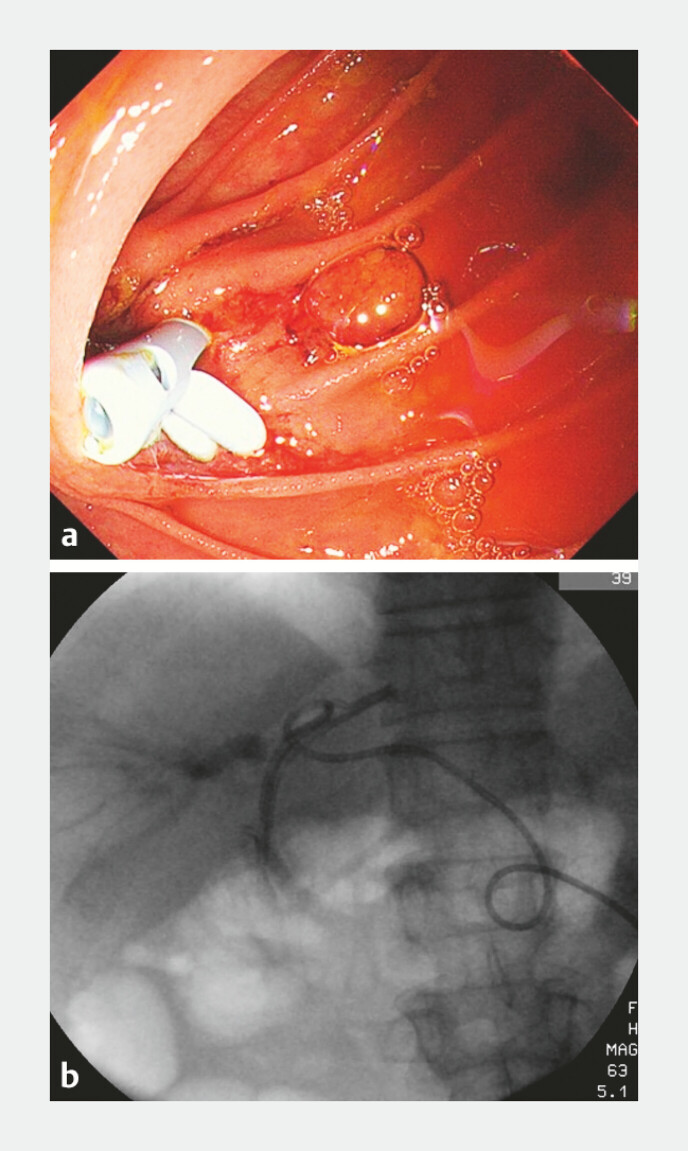
Two biliary stents were successfully placed: **a** endoscopic images and **b** radiological images.

A 51-year-old man with a history of pancreaticoduodenectomy was scheduled to undergo endoscopic retrograde cholangiopancreatography (ERCP) with biliary stent placement for biliary-enteric anastomotic strictures. During the ERCP procedure, the endoscope was smoothly advanced to the region of the biliary-enteric anastomosis; however, the anastomotic stoma could not be located ( Fig. 1 ). It was suspected that the biliary-enteric anastomosis might be situated behind the mucosal folds, making it invisible under the standard endoscopic view. A peroral cholangiopancreatoscope (9 Fr) was then introduced for further exploration ( Fig. 2 ). A pinhole-like anastomotic stoma was visualized behind the mucosal folds using a cholangiopancreatoscope, and the guidewire (0.035 in) was successfully inserted into the bile duct via this opening ( Fig. 3 ). Then, the cholangiopancreatoscope was removed for further steps using a standard scope ( Video 1 ). A needle knife was used to perform a 1–2 mm precut of the stoma ( Fig. 4 ). Subsequently, an 8-mm diameter dilation balloon was employed to dilate the biliary-enteric anastomosis smoothly, followed by the successful placement of two plastic biliary stents ( Fig. 5 ). The biliary stents were removed 8 months postoperatively. A follow-up examination 1 year after surgery showed no intrahepatic biliary dilation and no stricture at the biliary-enteric anastomosis.

Successful cannulation of biliary enteric anastomosis stenosis using a peroral cholangiopancreatoscope.Video 1

ERCP-guided biliary stent placement is the preferred treatment modality to patients with benign biliary strictures 1 . In this case, the biliary-enteric anastomosis was located behind the mucosal folds, which was beyond the effective visual field. The authors innovatively utilized the peroral cholangiopancreatoscope, taking advantage of its excellent passability in narrow spaces and its capacity to expand the visual field. The probe navigated around the mucosal folds, ultimately facilitating successful cannulation. Thanks to its slender scope and real-time visualization capability, peroral cholangiopancreatoscopy has been further expanded to ERCP cannulation by some scholars, who have consequently developed ERDC technology 2 , enabling radiation-free visualized precision cannulation.

Endoscopy_UCTN_Code_CCL_1AZ_2AZ Endoscopy_UCTN_Code_TTT_1AR_2AL Endoscopy_UCTN_Code_TTT_1AR_2AG
